# Supplementary material for: A tectonically driven Ediacaran oxygenation event
Source: Nat Commun. 2019 Jun 19;10:2690. doi: 10.1038/s41467-019-10286-x (PMC6584537; doi:10.1038/s41467-019-10286-x)
Supplement: Supplementary file 1 — Supplementary Information [file 41467_2019_10286_MOESM1_ESM.pdf]

Supplementary Information

A Tectonically Driven Ediacaran Oxygenation Event

Williams et al.

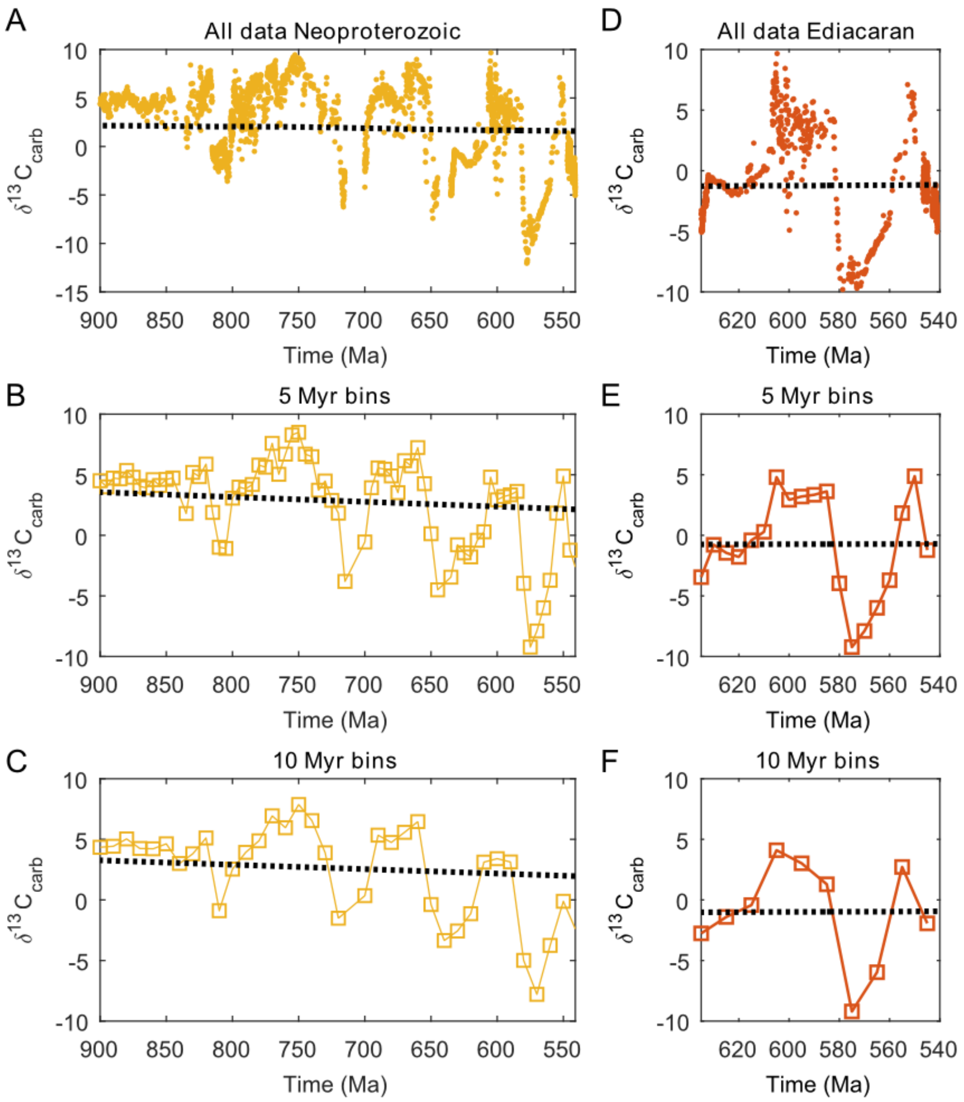

Supplementary Figure 1: *Least-squares regression analysis of carbonate Carbon isotope data [1] across the Neoproterozoic and Ediacaran. (A) All data across the Neoproterozoic. (B) Neoproterozoic data in 5 Myr bins. (C) Neoproterozoic data in 10 Myr bins. (D - F) Ediacaran data following the same binning procedure.*

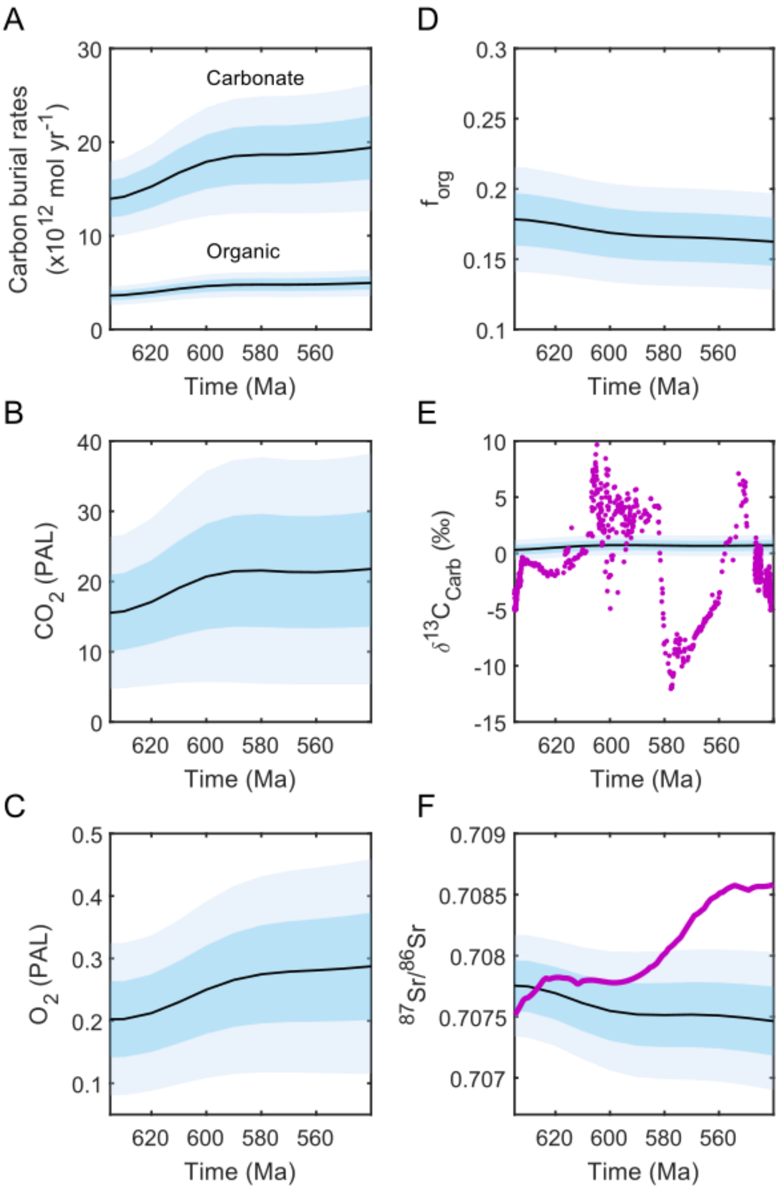

Supplementary Figure 2: *Monte-Carlo experiments running the COPSE model for the Ediacaran period. Degassing rates are sampled from the error window of Mills et al. (2017) [2], uplift rates are set constant at 1. Important parameters are chosen for each run from the ranges shown in table 1 in the main manuscript. Shaded windows show ±1 std. dev. (light) and ±0.5 std. dev. (dark). Geologic data in purple [1, 3]. (A) Carbon burial rates. (B) CO2, (C) O2, (D)* f_{org}*, (E)* $\delta^{13}C_{carb}$*, (F)* $^{87}Sr/^{86}Sr$*.*

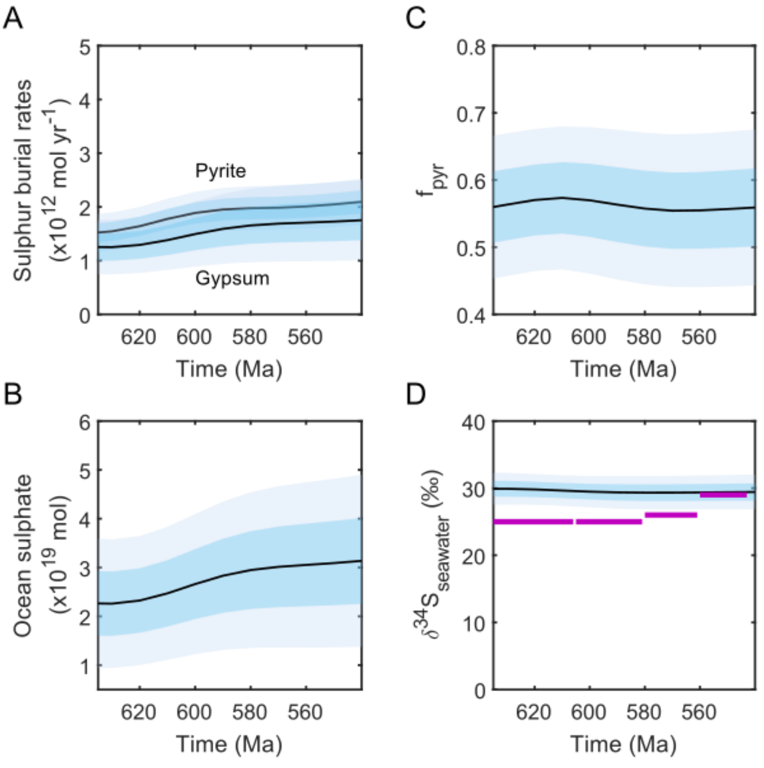

Supplementary Figure 3: *Sulphur cycling from the Monte-Carlo experiments where uplift is set constant to 1. Shaded windows show ±1 std. dev. (light) and ±0.5 std. dev. (dark). Geologic data in purple (averages of [4]). (A) Sulphur burial rates. (B) Ocean sulphate concentration, (C)* f_{py}*, (D)* $\delta^{34}S$ *of seawater.*

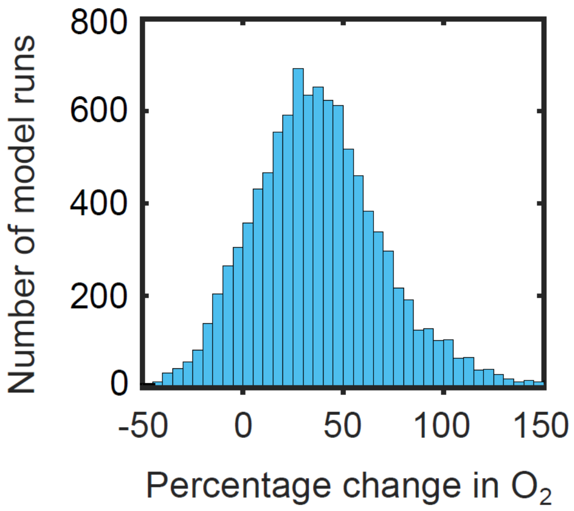

Supplementary Figure 4: *Probability distribution of percentage O_2 reservoir change between 640-620 Ma and 560-540 Ma under constant uplift. The mean O_2 value is taken from each age range.*

Supplementary Note 1: Full Monte-Carlo simulations under a constant uplift rate.

Here we present model outputs from a Monte Carlo simulation of 10,000 runs in which we keep the uplift forcing factor constant at 1 (Supplementary Figures 2 - 4). The rest of the simulation follows that described in the main manuscript. The key differences in these model outputs are the larger increase in CO_2 concentration (as uplift drives enhanced silicate weathering), and the greatly reduced fit to the Sr isotope record (as uplift drives the weathering of more radiogenic materials). We show this experiment to make it clear that our proposed mechanism and the resulting O_2 rise does not depend on the assumption of a large increase in uplift rates. O_2 rise in this model is very similar to that of the full model. This is because whilst increases in uplift and erosion act to increase the weathering of sedimentary organic carbon (an O_2 sink), they also act to increase other weathering fluxes which supply phosphorus and stimulate organic carbon and pyrite sulphur burial. Plots for the sulphur cycle (Supplementary Figure 3) show a slightly increasing rate of gypsum burial relative to pyrite burial throughout the run, but not a substantial difference in $\delta^{34}S$ when compared to the run in the manuscript in which uplift increases. Increasing uplift drives more pyrite burial than gypsum burial in the model by increasing both sulphate supply and nutrient supply to the oceans. Supplementary Figure 4 shows a probability histogram for the modelled increase in atmospheric O_2 between 640-620 Ma and 560-540 Ma. Approximately 88% of model runs continue to show an increase in atmospheric O_2.

Supplementary Table 1: Core COPSE model reservoirs and differential equations.

*denotes reservoirs that have been updated in this study.

**Reservoir**	**Label**	**Differential equation**	**Initial size (mol)**
Ocean (reactive) nitrogen	***N***	$\frac{d\boldsymbol{N}}{dt} = nfix - denit - monb$	4.35×10^{16}
Ocean (phosphate) phosphorus	***P***	$\frac{d\boldsymbol{P}}{dt} = psea - mopb - fepb - capb$	3.1×10^{15}
Atmosphere-ocean O_2*	***O***	$\frac{d\boldsymbol{O}}{dt} = locb + mocb - oxidw - ocdeg + 2 \cdot mpsb - 2 \cdot pyrw - 2 \cdot pyrdeg - rgf$	3.7×10^{19}
Atmosphere-ocean CO_2*	***A***	$\frac{dA}{dt} = oxidw + ocdeg + carbw + ccdeg + rgf - locb - mocb - mccb - sfw$	3.193×10^{18}
Sedimentary organic (reduced) carbon*	***G***	$\frac{d\boldsymbol{G}}{dt} = 0$	1.25×10^{21}
Sedimentary carbonate (oxidised) carbon*	***C***	$\frac{d\boldsymbol{C}}{dt} = 0$	5.0×10^{21}
Ocean (sulphate) sulphur	***S***	$\frac{d\boldsymbol{S}}{dt} = gypw + pyrw + gypdeg + pyrdeg - mgsb - mpsb$	4.0×10^{19}
Sedimentary pyrite (reduced) sulphur*	***PYR***	$\frac{d\boldsymbol{PYR}}{dt} = 0$	1.8×10^{20}

Sedimentary gypsum (oxidised) sulphur*	***GYP***	$\frac{d\boldsymbol{GYP}}{dt} = 0$	$2.0\text{x}10^{20}$

Supplementary Table 2: Core COPSE model fluxes and baseline values.

Process	Label	Constant	Present day value	Notes
Nitrogen cycle			$\text{x}10^{12}$ mol N yr^{-1}	
Nitrogen fixation	*Nfix*	k_3	8.67	
Denitrification	*Denit*	$2k_4$	8.6	
Marine organic N burial	*Monb*	k_2/CN_{sea}	0.07	CN_{sea}=37.5
Phosphorus cycle			$\text{x}10^{9}$ mol P yr^{-1}	
Reactive P weathering	*Phosw*	k_{10}	42.5	
Terrestrial organic P burial	*Pland*	$k_{11}k_{10}$	2.5	CP_{land}=1000
Reactive P to ocean	*Psea*	$(1-k_{11})k_{10}$	40	
Marine organic P burial	*Mopb*	k_2/CP_{sea}	10	CP_{sea}=250
Iron-sorbed (Fe-P) burial	*Fepb*	k_6	10	
Ca-bound (Ca-P) burial	*Capb*	k_7	20	
Carbon cycle (inorganic)			$\text{x}10^{12}$ mol C yr^{-1}	
Carbonate C degassing	*Ccdeg*	k_{12}	15	
Carbonate weathering	*Carbw*	k_{14}	8	

Silicate weathering	*Silw*	k_{silw}	12	
Granite weathering	*Granw*	k_{granw}	9	
Basalt weathering	*Basw*	k_{basw}	3	
Seafloor weathering	*Sfw*	k_{sfw}	3	
Carbonate burial	*Mccb*	$k_{14}+k_{silw}$	20	
Carbon cycle (organic)			x10^{12} mol C yr^{-1}	
Organic C degassing	*Ocdeg*	k_{13}	1.25	
Oxidative C weathering	*Oxidw*	k_{17}	3.75 – *rgf*	
Marine organic C burial	*Mocb*	k_2	2.5	
Terrestrial organic C burial	*Locb*	k_5	2.5	
Sulphur cycle			x10^{12} mol S yr^{-1}	
Gypsum degassing	*gypdeg*	k_{gypdeg}	0.5	
Pyrite degassing	*pyrdeg*	k_{pyrdeg}	0.25	
Gypsum weathering	*Gypw*	k_{22}	2.0	
Pyrite weathering	*Pyrw*	k_{21}	0.45	
Gypsum burial	*Mgsb*	k_{mgsb}	2.5	
Pyrite burial	*Mpsb*	k_{mpsb}	0.7	

Supplementary Table 3: Model forcing factors. (All normalised to 1 at present with the exception of insolation.)

*denotes forcing factors updated in this study

**denotes forcing factors set to constant values of 1 for this study

Forcing	Description	Basis	Source(s)
Original model forcing factors			
*D**	Metamorphic and volcanic degassing	Subduction Zone Length	2
*U**	Tectonic uplift	Model fit to $^{87}Sr/^{86}Sr$ data	-
CP_{land}	C/P burial ratio of terrestrial plant material	Attempt to capture Paleozoic coal deposition	5, 6
B	Apportioning of carbonate burial between shallow and deep seas	Fossil record of evolution of planktonic calicifiers	7
Additional forcing factors included herein			
a_{bas}**	Exposed area of volcanic silicate rocks	Reconstructed area of large igneous provinces (LIPs) and volcanic islands	8, 9
a_{gran}**	Kinetically-weighted area of non-volcanic silicate rocks	Reconstructed lithology of shield, shale, coal, evaporite	10
*PG***	Paleogeography effect on runoff/weathering	Climate model simulations	11
c_{cal}**	Ocean calcium concentration	Best fit to fluid inclusion data	12

Supplementary Table 4: COPSE model non-flux parameters. Flux parameters are in Supplementary Table 2. References and justification for chosen values are given in Lenton et al.[6].

Label	Meaning	Default value
k_1	Present oxic fraction	0.997527
k_{15}	Pre-plant weathering	0.15
k_{fire}	Fire frequency control	3
k_c	Climate sensitivity control	4.328°C
k_l	Luminosity sensitivity control	7.4°C
k_T^{sfw}	Temperature sensitivity of seafloor weathering	0.0608
k_T^{gran}	Temperature sensitivity of granite weathering	0.0724
k_T^{bas}	Temperature sensitivity of basalt weathering	0.0608
k_{Psilw}	Silicates fraction of P weathering	0.8
k_{Pcarbw}	Carbonates fraction of P weathering	0.14
k_{Poxidw}	Oxidative fraction of P weathering	0.06
k_{aq}	Terrestrial organic matter burial fraction in aquatic settings	0.8
k_u	Nutrient utilisation efficiency	0.5

k_{anox}	Sharpness of oxic-anoxic transition	12

Supplementary Table 5. Strontium reservoirs and differential equations.

Reservoir	Label	Differential equation	Present size (mol)
Ocean Sr	OSr	$\frac{dOSr}{dt} = Sr_{granw} + Sr_{basw} + Sr_{sedw} + Sr_{mantle} - Sr_{sedb} - Sr_{sfw}$	1.2×10^{17}
Sed. Sr	SSr	$\frac{dSSr}{dt} = Sr_{sedb} - Sr_{sedw} - Sr_{metam}$	5×10^{18}

Supplementary Table 6: Strontium fluxes and baseline (present day) values.

Process	Label	Constant	Baseline flux (molSr yr^{-1})	Source/Notes
(Silicate weathering)		k_{Srsilw}	13×10^{9}	13
Basalt weathering	Sr_{basw}	$k_{basfrac} \cdot k_{Srsilw}$		split follows carbon cycle
Granite weathering	Sr_{granw}	$(1-k_{basfrac}) \cdot k_{Srsilw}$		split follows carbon cycle
Sediment weathering	Sr_{sedw}	k_{Srsedw}	17×10^{9}	13
Mantle input	Sr_{mantle}	$k_{Srmantle}$	7.3×10^{9}	13
Seafloor weathering	Sr_{sfw}	k_{Srsfw}		split follows carbon cycle
Sediment burial	Sr_{sedb}	k_{Srsedb}		split follows carbon cycle

Sediment metamorphism	Sr_{metam}	$k_{Srmetam}$	13x10^9	13

Supplementary Table 7: Other constants in the strontium cycle.

Constant	Label	Value	Source/Notes
^{87}Rb decay rate	λ	1.4x10^{-11} yr^{-1}	13
Original value	$^{87}Sr/^{86}Sr_0$	0.69898	at formation of the Earth
$^{87}Sr/^{86}Sr$ basalt	δSr_{basalt}	0.705	13
$^{87}Sr/^{86}Sr$ granite	$\delta Sr_{granite}$	0.716	reproduces present ocean composition
$^{87}Sr/^{86}Sr$ mantle	δSr_{mantle}	0.703	13
$^{87}Rb/^{86}Sr$ mantle	$^{87}Rb/^{86}Sr_{mantle}$	0.066	for correct present day $^{87}Sr/^{86}Sr$
$^{87}Rb/^{86}Sr$ basalt	$^{87}Rb/^{86}Sr_{basalt}$	0.1	for correct present day $^{87}Sr/^{86}Sr$
$^{87}Rb/^{86}Sr$ granite	$^{87}Rb/^{86}Sr_{granite}$	0.26	for correct present day $^{87}Sr/^{86}Sr$
$^{87}Rb/^{86}Sr$ sediments	$^{87}Rb/^{86}Sr_{carbonate}$	0.5	for correct present day $^{87}Sr/^{86}Sr$ assuming crustal average $^{87}Sr/^{86}Sr$ of 0.73

Supplementary References:

[1] Saltzman, M.R. & Thomas, E. (2012) Chapter 11 - Carbon Isotope Stratigraphy in *The Geologic Time Scale*, edited by Felix M. Gradstein, James G. Ogg, Mark D. Schmitz, & Gabi M. Ogg (Elsevier, Boston, pp. 207-232.

[2] Mills, B.J.W., Scotese, C.R., Walding, N.G., Shields, G.A. and Lenton, T.M. (2017) Elevated CO_2 degassing rates prevented the return of Snowball Earth during the Phanerozoic, *Nature Communications*, **8**, doi:10.1038/s41467-017-01456-w.

[3] Cox, G.M., Halverson, G.P., Stevenson, R.K., Vokaty, M., Poirier, A., Kunzmann, M., Li, Z-X., Denyszyn, S.W., Strauss, J.V. and MacDonald, F.A. (2016) Continental flood basalt weathering as a trigger for Neoproterozoic Snowball Earth, *Earth and Planetary Science Letters*, **446**, p. 89-99.

[4] Canfield, D.E. and Farquhar, J. (2009) Animal evolution, bioturbation, and the sulfate concentration of the oceans, *Proceedings of the National Academy of Sciences*, **106(20)**, p. 8123-8127.

[5] Lenton, T.M., Dahl, T.W., Daines, S.J., Mills, B.J.W., Ozaki, K., Saltzman, M.R. and Porada, P. (2016) Earliest land plants created modern levels of atmospheric oxygen, *Proceedings of the National Academy of Sciences*, **113(35)**, p. 9704-9709.

[6] Lenton, T.M., Daines, S.J. and Mills, B.J.W. (2018) COPSE reloaded: An improved model of biogeochemical cycling over Phanerozoic time, *Earth-Science Reviews*, **178**, p. 2-18.

[7] Berner, R.A. (1994) GEOCARB II: A revised model of atmospheric CO2 over Phanerozoic time, *American Journal of Science*, **294**, p. 56-91.

[8] Mills, B.J.W., Daines, S.J. and Lenton, T.M. (2014) Changing tectonic controls on the long-term carbon cycle from Mesozoic to present, *Geochemistry, Geophysics, Geosystems*, **15(12)**, p. 4866-4884 (2014).

[9] Ernst, R.E. (2014) *Large Igneous Provinces*. Cambridge University Press, Cambridge, UK.

[10] Bluth, G.J.S. and Kump, L.R. (1991) Phanerozoic paleogeology, *American Journal of* Science, **291(3)**, p. 284-308.

[11] Royer, D.L., Donnadieu, Y., Park, J., Kowalczyk, J., and Godderis, Y. (2014) Error analysis of CO_2 and O_2 estimates from the long-term geochemical model GEOCARBSULF, *American Journal of Science*, **314**, p. 1259-1283.

[12] Horita, J., Zimmermann, H. and Holland, H.D. (2002) Chemical evolution of seawater during the Phanerozoic: Implications from the record of marine evaporites, *Geochimica et Cosmochimica Acta*, **66(21)**, p. 3733-3756.

[13] Francois, L.M. and Walker, J.C.G. (1992) Modelling the Phanerozoic carbon cycle and climate: Constraints from the $^{87}Sr/^{86}Sr$ isotopic ratio of seawater, *American Journal of Science*, **292**, p. 81-135.
